# Confining the growth of mesoporous silica films into nanospaces: towards surface nanopatterning†

**DOI:** 10.1039/d1na00654a

**Published:** 2022-01-11

**Authors:** Samantha Soulé, Gilles E. Moehl, Ruomeng Huang, Yasir J. Noori, Kian Shen Kiang, C. H. Kees de Groot, Richard Beanland, David C. Smith, Andrew L. Hector

**Affiliations:** Université de Lorraine, CNRS, LCPME 54600 Villers-lès-Nancy France samantha.soule@univ-lorraine.fr; School of Chemistry, University of Southampton Southampton SO17 1BJ UK A.L.Hector@soton.ac.uk; School of Electronics and Computer Science, University of Southampton Southampton SO17 1BJ UK; Department of Physics, University of Warwick Coventry CV4 7AL UK; School of Physics and Astronomy, University of Southampton Southampton SO17 1BJ UK

## Abstract

The combination of lithographic methods and sol gel bottom-up techniques is a promising approach for nanopatterning substrates. The integration and scalable fabrication of such substrates are of great interest for the development of nanowire-based materials opening potentialities in new technologies. We demonstrate the deposition of ordered mesoporous silica into nanopatterned silica substrates by dip coating. Using scanning electron microscopy and grazing incidence small angle X-ray scattering, the effect of the sol composition on the pore ordering was probed. Optimising the sol composition using anodic alumina membranes as confined spaces, we showed how the pH controlled the transformation from circular to columnar mesophase. Vertical mesopores were obtained with very good repeatability. The effect of the sol chemistry on the surfactant curvature was then shown to be similar in nanopatterned substrates made by e-beam lithography.

## Abstract 2

Mesoporous silica was deposited into e-beam patterned substrates and mesochannel orientation was controlled into nanospaces by tuning the sol chemistry opening potentialities in the fabrication of nanowire-based devices.

## Graphical abstract

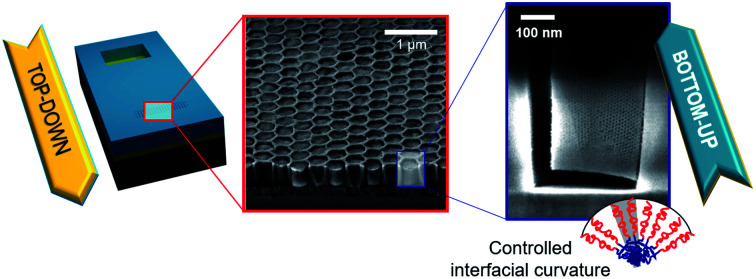

## Introduction

Surface patterning has attracted growing interest in semiconductor technology since miniaturising objects down to the nanoscale can improve device efficiency. Producing well-ordered surface patterns can be achieved by using top-down techniques such as lithography or scanning probe-based writing techniques.^1,2^ However, these methods often imply high cost fabrication to get high-resolution pattern design, and scanning probe methods are time-consuming meaning throughput is low.

The bottom-up approach is a promising alternative to produce high quality patterns at the nanoscale. The strategy consists of periodically self-assembling molecules or nano-objects into nanostructured materials from solution.^3^ Particularly, the combination of sol–gel and supramolecular chemistry leads to the formation of mesostructured materials with well-ordered and controlled porosity.^4^ Since the discovery of the co-assembly of silica-surfactant mesophases in 1992,^5^ research in this area has been extended to produce mesoporous materials with various compositions, pore structures and pore sizes. For thin film application, Brinker *et al.*^6^ developed the evaporation-induced self-assembly (EISA) process that has become the most versatile method to prepare mesoporous films. This method, often combined with dip coating as the deposition process, is based on the cooperative surfactant-silica self-assembly driven by solvent evaporation and leading to well-ordered mesoporous films. This process is very attractive considering the low cost equipment and easy processability but the resulting film often suffers from poor long-range order and the presence of localized points and extended defects.^7^

Combining lithographic methods and bottom-up sol–gel has started to emerge as a good strategy to produce complex hierarchical materials that could be integrated into real devices. However, as described by Innocenzi *et al.*^8^ patterning mesostructured films is very challenging. UV lithography^9^ or deep X-ray lithography^10^ are interesting to use on mesoporous films due to their modulable steady state^11^ allowing the change or disruption of the mesophase through the external radiation source. However, processing the film after deposition does not enable the precise patterning of a single well-ordered domain. Pre-patterning the surface using top-down techniques is most promising to obtain long-range ordered films. For example, patterned substrates prepared by micromoulding in capillaries^12^ (MIMIC) or e-beam lithography^13^ have been successfully used for the deposition of mesoporous silica films by EISA leading to oriented channels over a macroscopic scale. Wu *et al.*^13^ have shown strictly aligned mesochannels running parallel with identified nanodomains in 0.5 μm wide patterns (resist thickness 0.5 μm) obtained by spin coating. By decreasing the width of the patterned trenches to 0.1 μm, cross-sectional HRSEM images before resist removal and calcination showed mesochannels aligned either perpendicular or parallel to the substrate. Having mesoporous silica with a vertical alignment of mesopores and defined positions across a macroscopic substrate would be an ideal template for the development of nanowire-based devices.

On the other hand, Bolger *et al.*^14^ were not able to get aligned mesopores in patterned Si substrates when using spin coating as deposition method. They demonstrated that the directional flow of the sol during dip coating onto patterns was essential to get pore ordering with the trench guiding the alignment of the mesopores. The discrepancy between these two studies emphasises the importance of the experimental conditions. The evaporation induced self-assembly process is very sensitive to chemical conditions as well as the evaporation conditions (temperature, humidity).^11^ The chemical and physical phenomena occurring during evaporation are even more complex when ordered silica arrays are prepared in patterned substrates.

The growth of mesoporous silica in confined spaces has been specifically studied in anodic alumina membranes (AAM). The AAM commercial templates are characterised by high aspect ratio channels with a diameter of hundreds nanometers and a thickness of tens of microns. Yamaguchi *et al.* were the first to demonstrate the formation of hexagonal mesopores oriented predominantly along the wall of the columnar alumina with CTAB cationic surfactant.^15^ Then, Lu *et al.* showed the possibility to obtain vertical mesochannels from Pluronic P123 surfactant.^16^ However, due to the confinement imposed by the channels, the formation of curved mesophases, such as the circular hexagonal structure, is usually observed and quite difficult to avoid when using a non-ionic surfactant. Different groups have shown that by playing with the processing conditions (temperature, relative humidity) as well as the sol chemistry (addition of inorganic salt, different precursor), they could drive a phase transformation from circular to columnar hexagonal and produce mesoporous silica film with mesochannels aligned with the wall of the AAM.^17–19^ These hard templates are however brittle and fragile, making it difficult to integrate them into devices.^20^ Moreover, obtaining a completely filled, gap-free hybrid membrane is still a challenge on the macroscopic scale.

To the best of our knowledge, the deposition of mesoporous silica into patterned nanopillars has only been reported once.^21^ A silica precursor solution with a 3D mesophase (using P123 as surfactant) was spin coated into a resist mould (1 μm thick) patterned by electron beam lithography with various feature sizes and shapes. The deposition in 2.5 μm cylinder pillars patterned led to the formation of 3D open silica mesopores. The small angle X-ray scattering (SAXS) pattern did not show any peak that the author could attribute to a discrete distribution of the patterns.

In this paper, we report the deposition of ordered mesoporous silica into patterned silica substrates produced by e-beam lithography. We studied the effect of the sol chemistry by first optimising its composition to obtain vertical mesopores in commercial AAM. To address the repeatability issues reported in the literature,^20^ we showed that controlling the hydrolysis/condensation rate of TEOS through the pH of the sol is effective to drive the formation of the columnar hexagonal mesophase. Considering the potential of combining top-down and bottom-up approaches, we examined the potential of using a patterned silica substrate in combination with the sol composition to drive the orientation of mesoporous silica. The patterned substrates we designed could then be integrated into real devices.

We demonstrated, using scanning electron microscopy (SEM) and grazing incidence small angle X-ray scattering (GI-SAXS), that even though the surfactant curvature may be controlled through the hydrolysis and condensation rate as in AAM, the deposition method, surface chemistry and aspect ratio of the patterns influenced the final orientation. We obtained mainly 2D-hexagonal mesopores oriented parallel to the substrates, but also observed evidence that higher aspect ratio features may drive perpendicular orientation.

## Experimental

The fabrication process of the nano-patterned substrate is illustrated in Fig. S1.† The fabrication started by depositing a 500 nm insulating SiO_2_ layer onto a 6 inch silicon wafer *via* reactive sputtering. A conducting TiN layer of 200 nm was subsequently deposited by sputtering. This was followed by sputtering a 500 nm thick of SiO_2_ layer. This SiO_2_ layer was then patterned *via* an e-beam lithography and a reactive ion etch process to form nanoscale holes with diameters of *ca.* 280 nm. After that, the wafer was diced into small chips (8 × 20 mm) for mesoporous silica deposition.

Depositions into confined spaces were done by adjusting an existing EISA-procedure.^17^ First, tetraethyl orthosilicate (2.08 g, 0.01 mol, TEOS, Sigma-Aldrich) was mixed with HCl (3 g of 0.2 mol dm^−3^ or 0.4 mol dm^−3^ HCl), H_2_O (1.8 g, 0.1 mol), and ethanol (5 ml) and heated at 60 °C for 1 h to accomplish acid-catalysed hydrolysis of the silica precursor. In another container, Pluronic P123 (750 mg, 0.13 mmol, PEO-*b*-PPO-*b*-PEO, *M*_n_ ≈ 5800, Sigma-Aldrich) dissolved in ethanol (15 ml). When cooled to room temperature, the respective TEOS- and surfactant containing solutions were combined and LiCl (85 mg, 2 mmol) was added.

The EISA solution was used either for drop-casting deposition on AAM to maximise the filling or dip-coating into patterned substrates. The AAM (Whatman, 13 mm diameter, 0.2 μm pores) was placed on a plastic ring and 80 μl of solution was spread on the AAM surface and left to dry while controlling relative humidity. The relative humidity was controlled either by using closed vessels containing saturated solutions of salts, or by flowing a controlled proportion of dry and water-saturated N_2_ in a closed chamber during and for the following 3 h after deposition. The sample was finally calcined with a heating rate of 0.5 °C min^−1^ for 10 h at 120 °C, 5 h at 220 °C and 5 h at 500 °C. Mesoporous silica films were deposited on patterned substrates by dip-coating (NIMA Technology 5.20 dip-coater) at constant withdrawal rate (60 mm min^−1^) while controlling relative humidity. Then the sample was aged for 3 days before being annealed at 120 °C for 10 h (0.5 °C min^−1^), then washed with ethanol for 2 h (Soxhlet extraction) and annealed again for 12 h using the same conditions.

In this work, the samples are named with capital letter M or S indicating the deposition on respectively membrane (AAM) or substrate. The HCl concentration is denoted 02 or 04 for using HCl 0.2 or 0.4 mol dm^−3^ giving a final HCl concentration in the sol of 0.022 or 0.044 mol dm^−3^. The relative humidity is indicated using the corresponding number (55 for 55% relative humidity). Added salt is given by its formula at the end of the name. For example, a sample named S-04-55-LiCl corresponds to a deposition in patterned substrate, at 55% relative humidity and using HCl 0.4 mol dm^−3^ and LiCl in the sol.

Small angle X-ray scattering measurements were recorded on a Rigaku Smartlab diffractometer with a parallel beam of Cu K_α_ X-rays and a HyPix 2D detector (0.3 mm collimator, sample-detector distance: 301 mm). The AAM were characterised using grazing-incidence transmission configuration. To determine the mesoporosity of the film into the patterns, the excess film above the patterns was removed by wiping the surface with absorbent paper just after the sample aging at room temperature. Then, after the subsequent surfactant removal by Soxhlet extraction, the substrate showed a clean surface (Fig. 3). GI-SAXS patterns were then collected with a 0.3° incidence angle. Data treatment (azimuthal integration, linecuts) were carried out using DPDAK software.^22^ The peak positions in the GI-SAXS pattern were calculated using GIXSGUI toolbox.^23^ The peak identified in all the GI-SAXS patterns at *q*_*z*_ = 1.35 nm^−1^ (see *q*_*z*_ linecuts in Fig. S5†) corresponded to the patterned substrates (TiN/SiO_2_) we used for the deposition. For the calculated patterns, the hollow circles and the squares corresponded to the transmissive and the reflective Bragg peaks respectively.

Cross section specimens were prepared for SEM by back thinning to ∼100 nm and cleaving, followed by Ar^+^ ion milling (Gatan PIPS ion mill) of the cleaved surface at 6 kV, with the ion beam incident from the substrate side only, at an angle of ∼2 degrees. Plan view sections were also obtained using Ar^+^ ion milling, with the ion beam ablating the top surface at a grazing incidence of 3 degrees while the sample rotated continuously. Scanning electron microscopy (SEM) was performed using a Zeiss Gemini SEM at ∼1 keV.

## Results and discussion

As most of the work available in literature was done in anodic alumina membranes (AAM), we first refined the sol composition by working with these commercial patterned substrates.

**Fig. 1:**
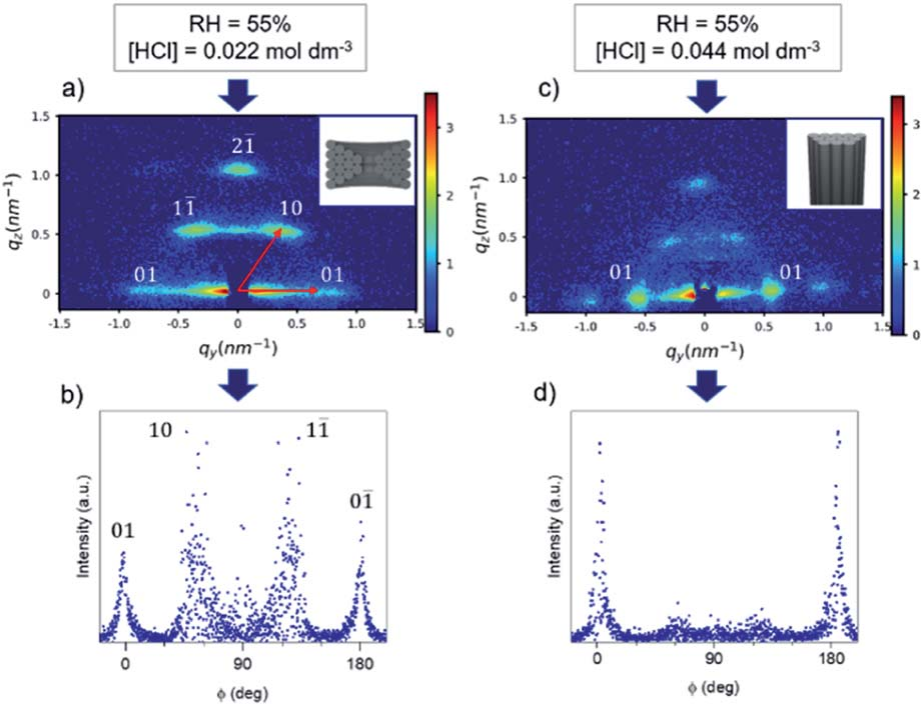
GT-SAXS patterns and the corresponding azimuthal integration of M-02-55-LiCl (a and b) and M-04-55-LiCl (c and d).

The initial deposition was done using an existing procedure^17^ (at 55% relative humidity (RH) and using HCl 0.2 mol dm^−3^ and LiCl in the sol) to obtain vertically aligned mesochannels. The GT-SAXS pattern of the sample referred as M-02-LiCl-55 (Fig. 1a), showed two bright out-of-plane (oop) signals (10 and 1−1 reflections) and two in-plane (ip) reflections (01 and 0−1), all corresponding to the circular mesophase. Because the columnar mesophase is also characterised by the 01 and 0–1 in-plane reflections, the oop : ip ratio must be evaluated to estimate semi-quantitatively the phase distribution in one sample. In this work, the determination of the oop : ip ratio was carried out using an azimuthal integration followed by fitting Gaussian to the resulting one-dimension peaks (Fig. 1b) and dividing the peak intensities. The oop : ip ratio derived from the pattern of M-02-LiCl-55 is 1 indicating a circular orientation (Table 1).

**Table 1:** Summary of data extracted from the GT-SAXS pattern of the different samples depending on the experimental conditions

Sample name	Relative humidity (RH, %)	[HCl] (mol dm^−3^)	*d* Spacing (nm)	oop : ip ratio
M-02-35-LiCl	35	0.022	11.3	0.5
M-02-55-LiCl	55	0.022	10.5	1
M-02-65-LiCl	65	0.022	10.8	1.4
M-04-35-LiCl	35	0.044	11.2	1
M-04-55-LiCl	55	0.044	11.3	<0.1
M-04-65-LiCl	65	0.044	11.3	0.4

As suggested by Platschek *et al.*,^24^ the formation mechanism of mesostructured silica in AAM using Pluronic P123 as surfactant started with random nucleation of a circular hexagonal mesophase (kinetically favoured) that partially transforms into a columnar phase with a more pronounced transformation at low humidity. We varied the relative humidity during the evaporation process to induce change in the micellar curvature of the surfactant (Fig. S2†). Decreasing the humidity to 35% lowered the oop : ip ratio which could be the result of the dehydration of the EO head-group area. However, none of the samples showed a perfect vertical alignment. Similar results were reported by Kurttepeli *et al.*^18^ with the same experimental conditions. In this procedure, the addition of LiCl in the sol was expected to induce a phase transformation from circular to columnar hexagonal.^24^ However, this effect is temperature dependent which may explain in our case the resulting circular mesophase.

**Fig. 2:**
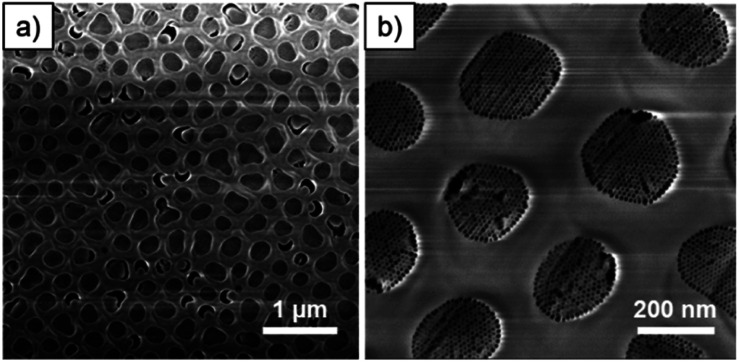
(a) Low and (b) high magnification SEM images of ion-milled M-04-55-LiCl.

As phase formation in block copolymer templated mesoporous powdered materials has been shown to be affected by the acidity,^25^ the HCl concentration was varied to understand its effect on the film porosity. The HCl concentration was increased in the starting sol from 0.022 to 0.044 mol dm^−3^. The GT-SAXS pattern of M-04-55-LiCl with the corresponding azimuthal integration are presented in Fig. 1c and d. Depositions were also carried at RH = 35% and 65% (GT-SAXS patterns and azimuthal integrations in Fig. S3†). The azimuthal integration in Fig. 1d clearly showed the decrease of the intensity associated with the oop reflections. The oop : ip ratios were significantly lowered for M-04-LiCl samples when the relative humidity was higher or equal to 55% (Table 1). For M-04-55-LiCl, we calculated a ratio lower than 0.1 demonstrating the formation of mainly vertical mesopores with a *d* spacing of 11.3 nm. The SEM images of M-04-55-LiCl (Fig. 2b) confirmed the well-organised columnar hexagonal mesophase with the mesoporous silica well attached to the channel surface (Fig. 2a).

By increasing the HCl concentration in the starting sol to 0.044 mol dm^−3^, we demonstrated the transformation from circular to columnar hexagonal. The mesophase orientation was controlled by adjusting the rate of mesophase silica precipitation with the surfactant species. In fact, the pH of the solution moved away from the isoelectric point of silica and the acid anion concentration increases. Consequently, the condensation kinetics of TEOS increased, decreasing the number of protonated silanol groups and finally led to a faster phase separation. As a result, the number of hydrogen bonding interactions with the EO blocks decreased, reducing further the swelling of EO blocks and leading to a decrease of the interfacial curvature.

Whereas we have only been able to decrease the proportion of the circular orientation with the combination of salting-out ions (chloride) and a controlled humidity, by modulating the acid concentration in the starting sol, we achieved the desired columnar hexagonal mesophase. We were able to repeat this result with very low variation in the proportion of the obtained columnar mesophase. Nevertheless, the filling of all the AAM channels as well as the brittleness of the membrane hinders its final use for applications.

Substrates patterned with cylindrical holes were then used to confine the growth of mesoporous silica. As Nealey *et al.*^26^ mentioned, the deposited structures are determined by the size and quality of the lithographically designed surface pattern rather than by the inherent limitations of the self-assembly process.

**Fig. 3:**
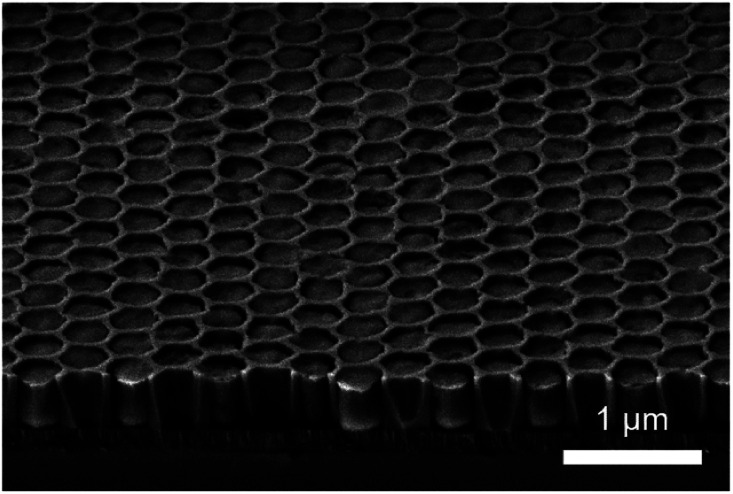
Cross-section SEM image of S-04-55-LiCl showing a clear surface and the complete filling of lithographical patterns.

Fabrication of densely packed nanoscale SiO_2_ holes with large aspect ratio can be challenging. In our case, the aspect ratio was limited to *ca.* 1 : 2 to ensure a uniform hole pattern and depth profile. The patterned substrates consisted of a close-packed hexagonal arrangement of holes (around 280 nm in diameter) etched in a 500 nm thick SiO_2_ layer. After removal of the excess of film and the surfactant, the cross-section SEM image showed patterns completely filled with mesoporous silica (Fig. 3).

**Fig. 4:**
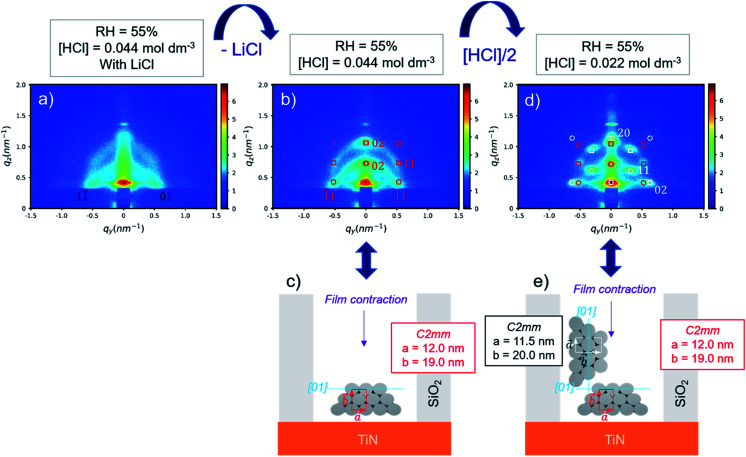
(a) GI-SAXS pattern of S-04-55-LiCl (indexed using *P*6*mm* as mesostructure); overlay of the GI-SAXS pattern with the simulated pattern and schematic representation of the corresponding pore arrangement and the cell parameters used to simulate the pattern of S-04-55 (b and c) and S-02-55 (d and e) (hollow circles: transmissive spots and hollow squares: reflective spots).

Dip coating was used as deposition method in patterned substrates considering the very high degree of accuracy over the deposition parameters and its high suitability to homogeneously impregnate patterns of such size.^27,28^ The GI-SAXS pattern of S-04-55-LiCl shown in Fig. 4a displays two main reflections corresponding to a 2D hexagonal arrangement of mesopores characterised by a *d* spacing of 10.1 nm. The orientation is difficult to assess from the GI-SAXS pattern. The absence (or very low intensity) of out-of-plane reflections (vertical linecut *q*_*z*_ in Fig. S5a†) could suggest the presence of perpendicular mesopores.

**Fig. 5:**
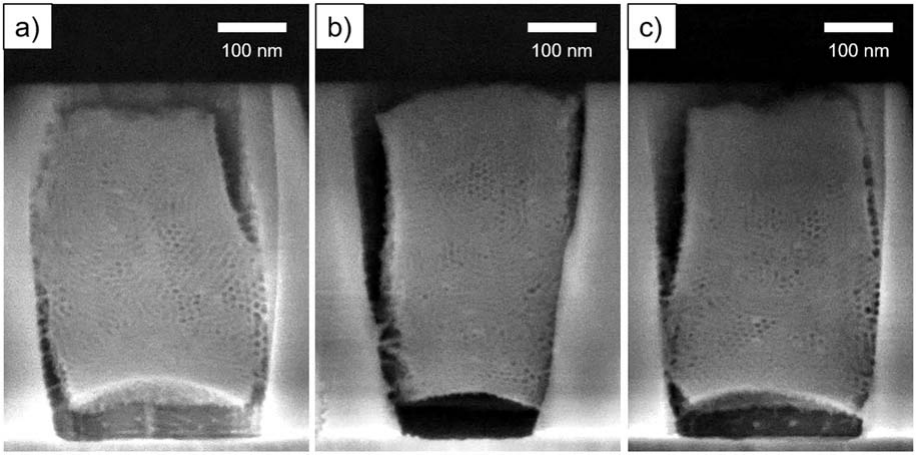
Cross section SEM images of ion-milled S-04-55-LiCl. Images (a–c) correspond to three different areas on the sample.

As can been seen in Fig. 3, the silica deposits remained intact after cleavage. The section was thus Ar^+^ ion-milled to reveal the porosity (Fig. S4a and b†). The cross-section SEM images (Fig. 5) displayed ordered mesopores with a hexagonal arrangement and different orientation. We observed nanodomains with mesopores parallel to the substrate and in some areas, mesopores seemed to run almost perpendicular (Fig. 5a). Shrinkage of the film happened at the interface with the TiN surface whereas the gap formation at the interface of the patterns was not systematic.

Besides the change in kinetics by modulating the pH, the addition of LiCl in the starting sol was necessary to induce the transformation from circular to columnar and to stabilise the formed mesostructure against shrinkage in the AAM channel.^17^ However, in SiO_2_ patterned substrates, we observed a dewetting phenomenon happening at some spots on the surface, sometimes leading to the formation of holes (Fig. S6†) in the mesoporous silica film. This may be the result of the LiCl salt increasing the surface tension of the sol solution.

By removing LiCl in the sol, the GI-SAXS pattern of deposited mesoporous silica changed (Fig. 4b). The pattern showed a clear out-of-plane reflection associated with a high intensity (*q*_*z*_ linecut in Fig. S5b†). This result corresponded to the formation of a 2D-hexagonal arrangement of pores oriented parallel to the substrate.

The indexation was done using GIXSGUI toolbox^23^ and considering the lattice as a distorted 2D-hexagonal mesophase^29^ (*C*2*mm* space group) due to the shrinkage happening in the direction normal to the substrate. The simulated positions calculated with *a* = 12.0 nm and *b* = 19.0 nm and the [01] direction parallel to the substrate as depicted in Fig. 4c were in good agreement with the experimental GI-SAXS pattern (Fig. 4b).

**Fig. 6:**
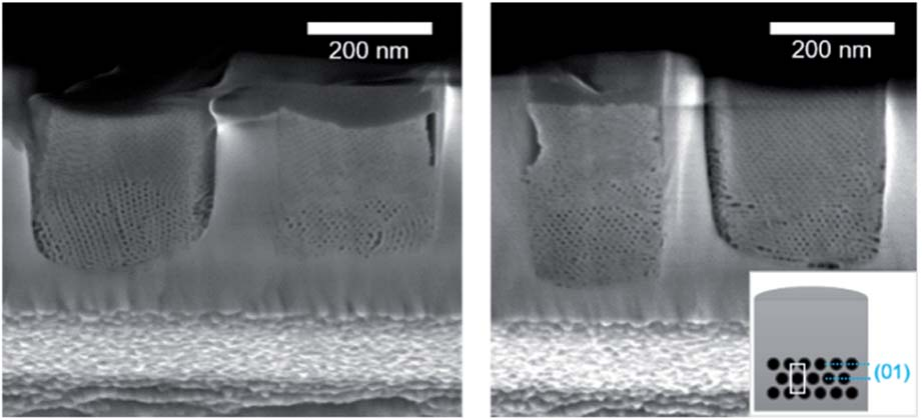
Cross-section SEM images of ion-milled S-04-55 (inset showing a diagram of the possible orientation of the mesochannels in confined cylindrical nanospaces).

As shown by SEM (Fig. 6), the film is well-attached to the pattern surfaces with few gaps at the interface. The cross-section images show the formation of the hexagonally packed mesochannels mainly from the bottom surface with the (01) plane parallel to the bottom plane in agreement with the GI-SAXS data. The mesoporous silica film did not show any defect in this case showing the influence of LiCl on the wetting properties of the sol solution. However, the comparison of S-04-55-LiCl and S-04-55 in Fig. 5 and 6 clearly demonstrated the disruption of the surfactant-silicate assembly by adding LiCl in the sol.

**Fig. 7:**
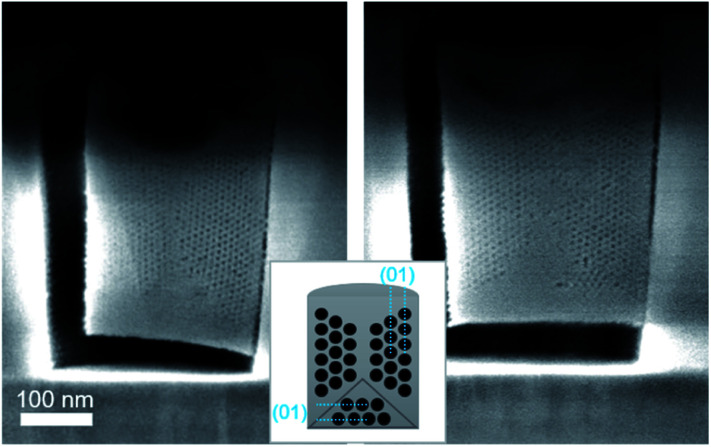
Cross-section SEM images after ion milling of S-02-55 (inset showing a diagram of the possible orientation of the mesochannels in confined cylindrical nanospaces).

When reducing the HCl concentration to 0.0022 mol dm^−3^ (sample S-02-55), the GI-SAXS pattern showed additional reflections (the white spots in Fig. 4d) compared to S-04-55. The cross-section SEM images (Fig. 7) revealed in each pattern the formation of mesoporous silica attached only from one side plane with different nanodomains of mesochannels. The first domain (noted D_⊥_) was characterised by a 2D-hexagonal arrangement of mesopores with the (01) plane perpendicular to the substrate. The other one (D_∥_) showed mesopores formed from the bottom surface with the (01) plane parallel to the substrate.

The simulation of a 2D-distorted hexagonal lattice with *a* = 11.5 nm, *b* = 20.0 nm and the [01] direction perpendicular to the substrate gave diffraction spots corresponding to the additional reflections observed (white spots in Fig. 4d). Therefore, the experimental GI-SAXS pattern in Fig. 4d was the superposition of two patterns corresponding both to a 2D-distorted hexagonal lattice of mesopores parallel to the substrate with D_⊥_ and D_∥_ nanodomains.

**Fig. 8:**
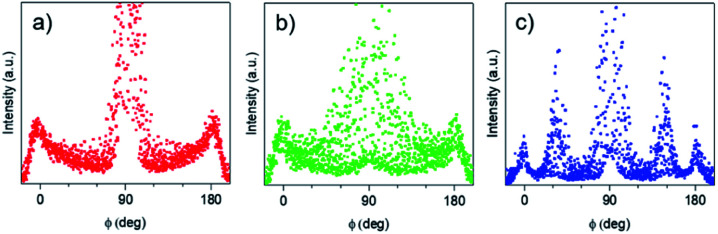
Azimuthal integrations of (a) S-04-55-LiCl, (b) S-04-55 and (c) S-02-55 GI-SAXS patterns.

The azimuthal integration (Fig. 8c) showed five peaks at 0°, 35°, 90°, 145° and 180°, which are the results of the two nanodomains. By comparison with S-04-55 (Fig. 8b), the peak at 0° corresponded to the superposition of the 11 reflected Bragg peak of D_∥_ and the 02 reflected peak of D_⊥_ (Fig. 4d) whereas the peak at 35° corresponded only to (11) reflection of D_⊥_. Comparing the intensities of these two peaks, the proportion of D_⊥_ appeared to be higher than D_∥_ in the S-02-55 sample. This result agreed with the SEM images in Fig. 7 showing only few mesopores growing from the substrate leading to D_∥_ nanodomain smaller than the D_⊥_.

The D_⊥_ nanodomain could correspond to a circular hexagonal mesophase. In fact, the combination of a low HCl concentration with no salt should lead to a high curvature interface into the confined nanospaces. This result further demonstrates that mesopore orientation can be driven by the sol composition while confining the growth of mesoporous silica into cylindrical nanopatterns. Additionally, the evaporation conditions as well as the patterns size, surface and quality will significantly affect the final orientation.

Using the same conditions as S-04-55 resulting in low surfactant curvature with higher aspect ratio cylindrical nanopatterns is likely to drive the mesopores perpendicular to the substrate, giving access to nanostructures with highly accessible mesopores. Such nanopatterns can be achieved in industry with advanced lithography and etching techniques. Different processes could also be considered for the design of such patterned lithographic substrates. As example, the use of novel self-assembled reactive nanomasks have shown promising results with the formation of nanopatterns with a 1 : 4 aspect ratio through dry etching.^30^

## Conclusions

In conclusion, we have demonstrated the control of the mesochannel orientation into nanospaces by modulating the sol chemistry. First, we have evidenced that adjusting the rate of mesophase silica precipitation with the surfactant species favours the columnar orientation. We proposed a reproducible experimental procedure leading to a high proportion of vertical mesopores in AAM by modulating the pH of the sol solution.

We have then showed the sol chemistry governed the surfactant curvature in the same manner in nanopatterned substrates made by e-beam lithography. For the first time, we reported the deposition of ordered mesoporous silica into patterned cylinders with pores oriented parallel. Whereas the addition of salting out anions in this case lead to a dewetting phenomenon in the film, the pH of the sol may be the critical parameter to control the orientation. The deposition into higher aspect ratio nanospaces is also required for the fabrication of mesoporous silica with vertical pores opening great potentialities in nanowire-based devices.

## Data availability

The data that support the findings of this study are openly available at the following URL/DOI: https:/doi.org/10.5258/SOTON/D2093.

## Conflicts of interest

There are no conflicts to declare.

## Supplementary Material

NA-004-D1NA00654A-s001
