# Supplementary material for: Confining the growth of mesoporous silica films into nanospaces: towards surface nanopatterning
Source: Nanoscale Adv. 2022 Jan 11;4(4):1105–11. doi: 10.1039/d1na00654a (PMC9417874; doi:10.1039/d1na00654a)
Supplement: NA-004-D1NA00654A-s001 [file NA-004-D1NA00654A-s001.pdf]

Electronic Supplementary Material (ESI) for Nanoscale Advances.
This journal is © The Royal Society of Chemistry 2022

Supporting Information

Confining the growth of mesoporous silica films into nanospaces: towards surface nanopatterning

Samantha Soulé, Gilles E. Moehl, Ruomeng Huang, Yasir J. Noori, Kian Shen Kiang, C. H. Kees de Groot, Richard Beanland, David C. Smith, Andrew L. Hector**

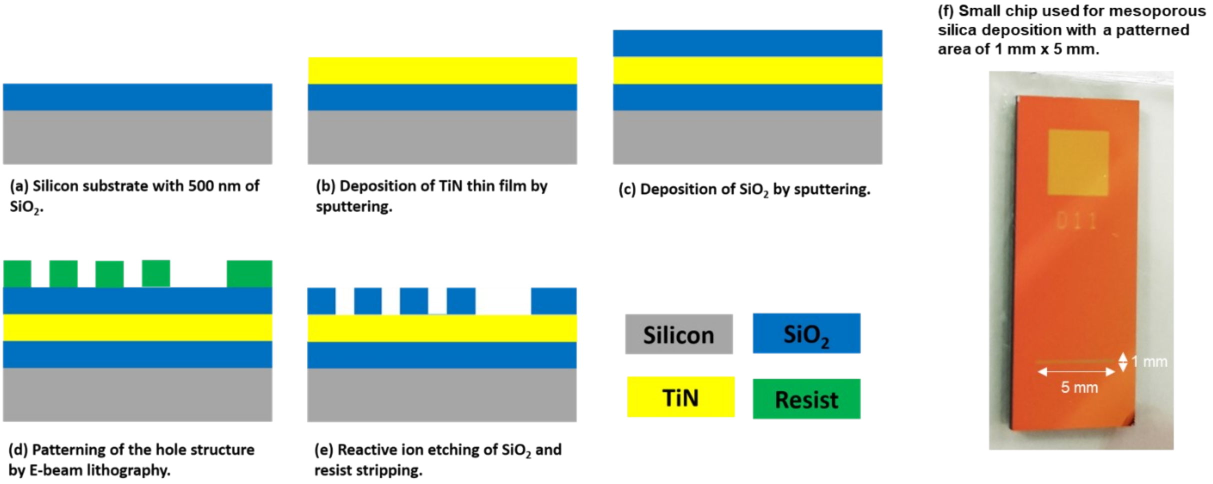

Figure S1- Fabrication process and photograph of the patterned substrate

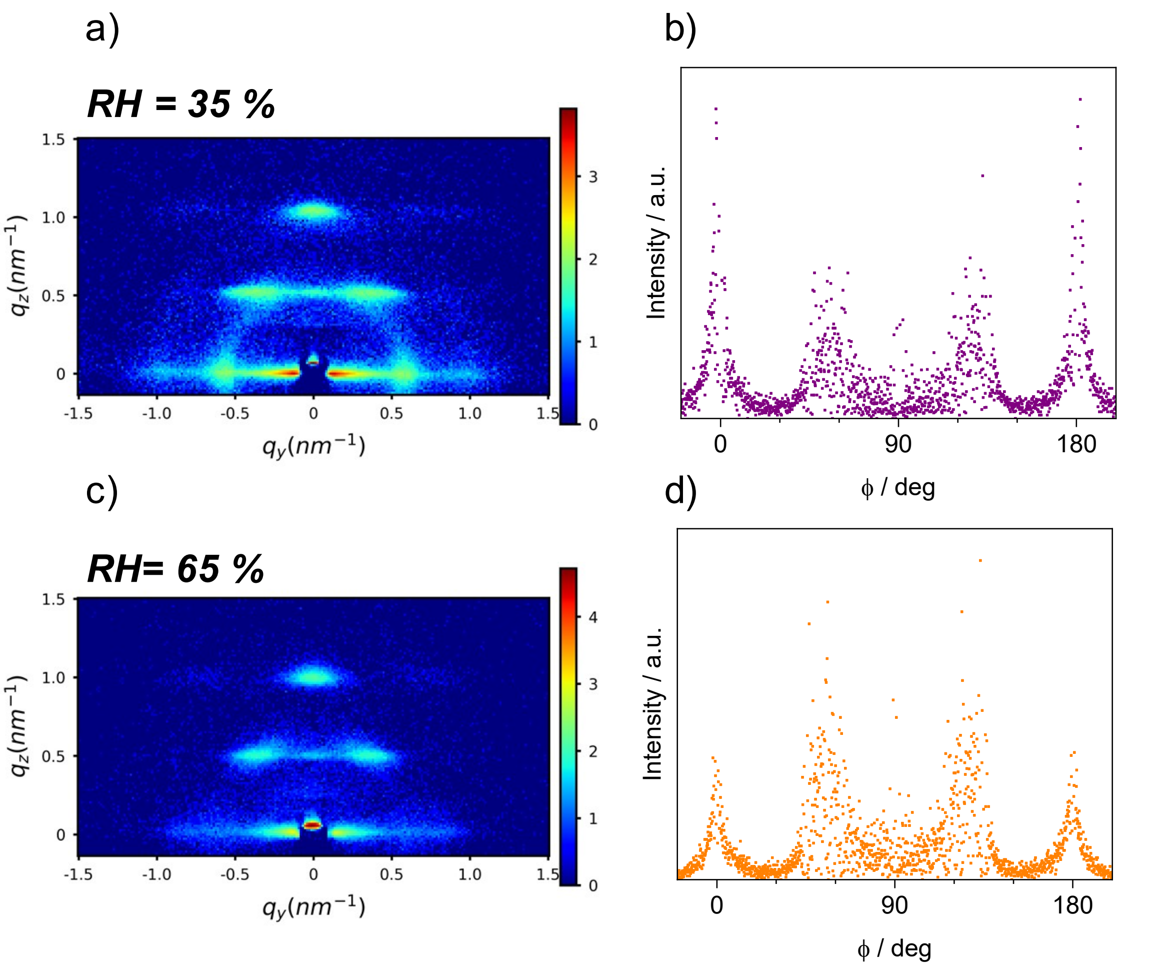

Figure S2- GT-SAXS patterns and the corresponding azimuthal integrations of M-02-35-LiCl (a, b) and M-02-65-LiCl (c, d)

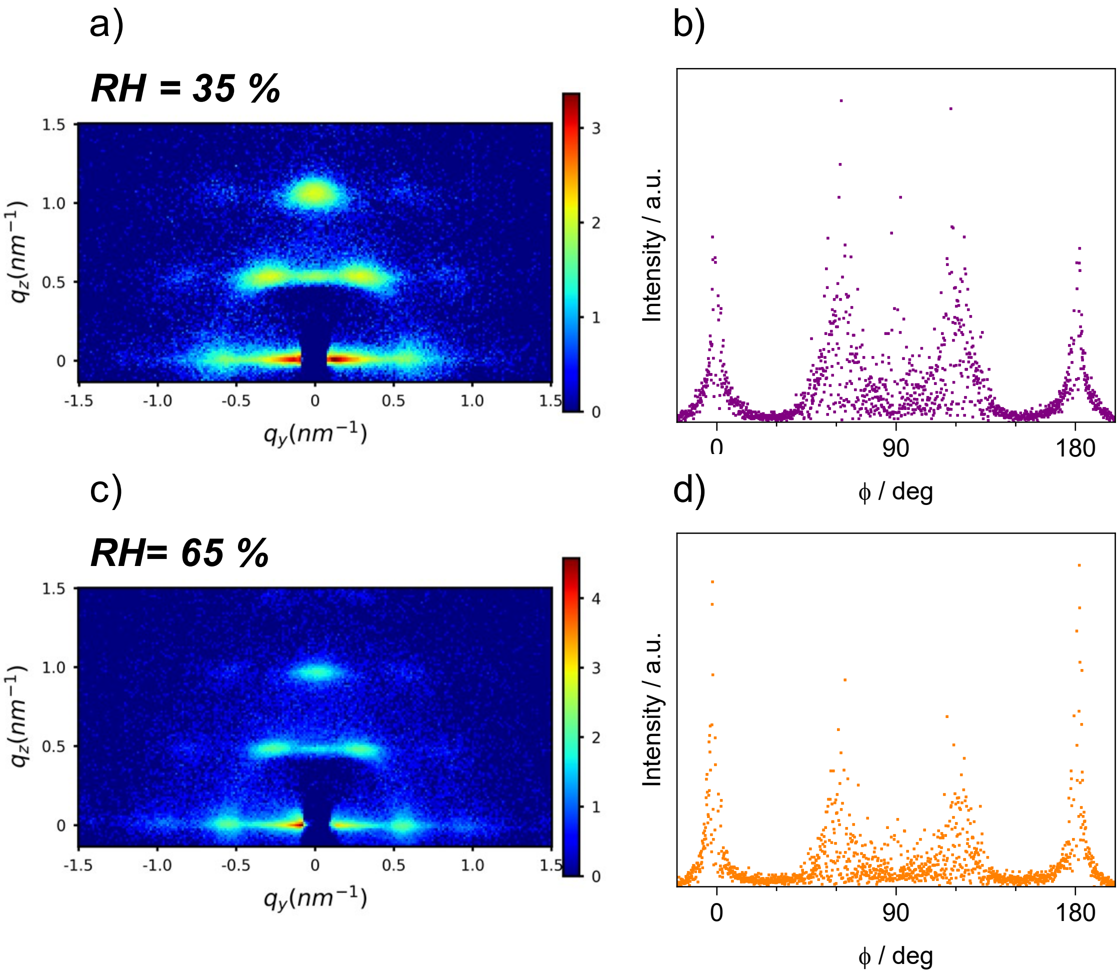

Figure S3- GT-SAXS patterns and the corresponding azimuthal integrations of M-04-35-LiCl (a, b) and M-04-65-LiCl (c, d)

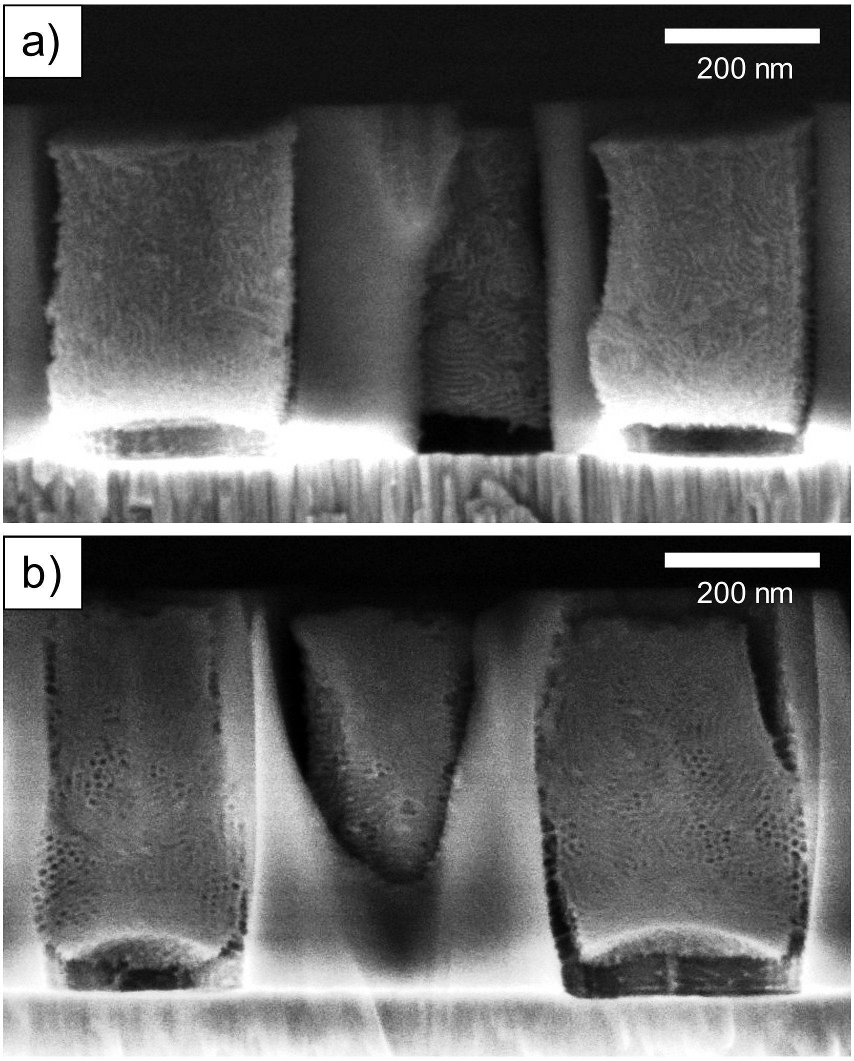

Figure S4- Cross section SEM images of S-04-55-LiCl a) as cleaved and b) ion-milled

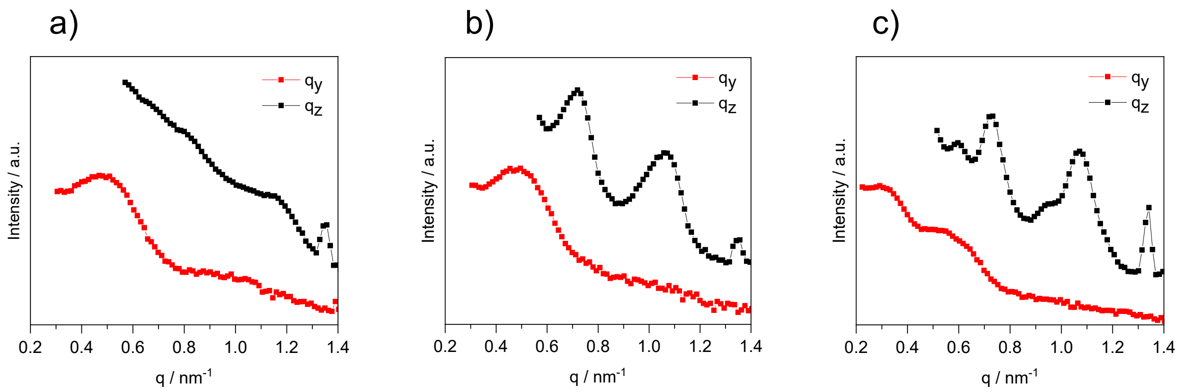

Figure S5- Horizontal (q_y) and vertical (q_z) linecut signals obtained from the GI-SAXS pattern of a) S-04-55-LiCl, b) S-04-55 and c) S-02-55

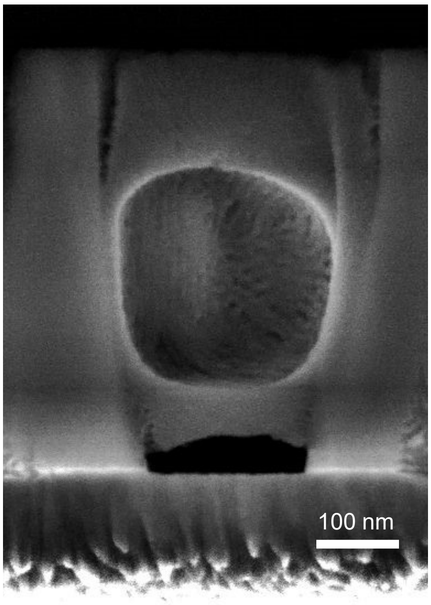

Figure S6- Cross-section SEM image of ion-milled S-04-55-LiCl evidencing a dewetting phenomenon leading to the formation of hole into the mesoporous silica film
